# Pareto-Driven Multiobjective Design of Axial-Flow Automotive Fan with Response Surface Modeling

**DOI:** 10.1021/acsomega.5c12213

**Published:** 2026-02-27

**Authors:** Kai Ren, Yuxi Chen, Guoqing Wang, Yujing Xu, Fei Yan, Min Dong

**Affiliations:** † School of Mechanical and Electronic Engineering, 74584 Nanjing Forestry University, Nanjing 210037, China; ‡ Nanjing Xiaoxun UAV Technology Co., Ltd, Nanjing 210037, China

## Abstract

Automotive cooling fans play a vital role in thermal management, yet conventional designs often struggle to balance efficiency, pressure, and flow requirements. This work presents a multiobjective optimization of an axial-flow fan using response surface methodology and a genetic algorithm. Four critical parameters (the root and tip installation angles and sweep angles) were optimized with respect to volumetric flow rate (*Q*), static pressure (*P*), and efficiency (η). A surrogate model built from 25 Latin Hypercube Sampling points achieved high accuracy (*R* ^2^ > 0.99). Sensitivity analysis showed that the tip angle predominantly affects *Q* and *P*, while the root angle strongly influences η. Optimization yielded Pareto solutions, where the efficiency improved from 18.31% to 21.19% without reducing the flow or pressure. The flow-field analysis demonstrated that the enhanced aerodynamic stability is addressed in the enhanced aerodynamic stability, characterized by smoother velocity profiles and reduced regions of separation and recirculation. The proposed framework not only improves fan aerodynamic efficiency but also establishes a generalizable strategy for systematic multiobjective optimization of rotating machinery.

## Graphical abstract

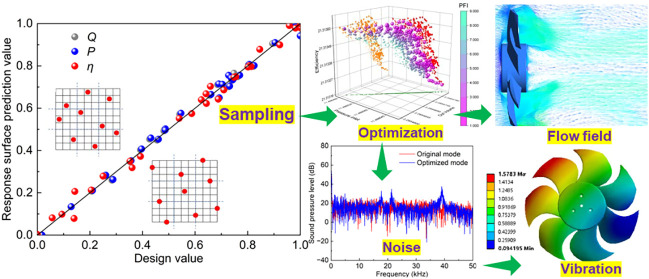

## 1 Introduction

With the continuous development of the automotive industry and increasing emphasis on energy conservation and emission reduction, engine thermal management has become increasingly critical. As the core system for maintaining the engine within the optimal operating temperature range, the performance of the cooling system not only affects the power output of the engine and fuel economy but also directly influences the reliability and service life of the vehicle.^1,2^ Among the critical components of the cooling system, axial-flow fans hold a central position due to their crucial role in regulating airflow and enhancing heat dissipation. These fans accelerate air along the axial direction through rotating blades, effectively directing external cold air through the radiator to dissipate heat absorbed by the coolant and thereby ensuring continuous engine cooling. Owing to their simple structure, high flow rate, energy efficiency, and low manufacturing cost, axial-flow fans have been widely adopted in vehicle cooling systems.^3,4^

However, with increasing demands for automotive performance, traditional experience-driven fan design has gradually proven inadequate in meeting the comprehensive performance requirements of modern vehicles in terms of cooling capacity, aerodynamic efficiency, and noise control.^5^ Consequently, how to systematically analyze and optimize the structural parameters of fans based on modeling and intelligent optimization methods has become a research focus in the fields of thermal management and mechanical design.^6,7^ In recent years, considerable strategies have been developed, including neural networks,^8^ evolutionary algorithms, high-throughput computing,^9,10^ and response surface methodology,^11–13^ to enhance design efficiency and performance for the fan. For instance, by combining artificial neural networks (ANN) with genetic algorithms (GA), the impeller blade has been significantly improved by static pressure and efficiency.^14^ A joint design system integrating deep neural networks and genetic algorithms has been explored, achieving improved design efficiency and effective cost control.^15^ An airfoil optimization method incorporating torsional constants into the objective function has been proposed, which can enhance the multiperformance adaptability of blade profiles.^16^ The response surface methodology, as a surrogate model, exhibits higher predictive efficiency and modeling accuracy in low-complexity data scenarios.^17^ The global optimization of an axial-flow fan by combining BP neural networks with genetic algorithms is addressed by achieving an efficiency improvement exceeding 10%.^18^ These investigations fully demonstrate the feasibility and effectiveness of integrating surrogate modeling methods with intelligent optimization algorithms to design the fan. The aerodynamic performance of fans is influenced by the coupled effects of multiple factors, including blade installation and sweep angles, inlet/outlet boundary conditions, rotational speed, and vehicle body structural layout.^19^ Under these influences, the internal flow of fans exhibits strong three-dimensional and unsteady characteristics, leading to complex phenomena such as flow separation, trailing vortices, backflow, and pressure fluctuations, which in turn affect their operational efficiency, noise levels, and stability.^20,21^ Therefore, constructing surrogate models and employing multiobjective optimization algorithms for systematic parameter optimization for the structure of the air-conditioning system cooling fan in automobile holds significant engineering values for improving aerodynamic performance and overall cooling effectiveness.

To address the abovementioned challenges, this study employs a Pareto-driven multiobjective optimization framework for an automotive axial-flow cooling fan by combining response surface methodology (RSM) with a multiobjective genetic algorithm (MOGA). A Latin hypercube sampling (LHS) scheme is used to generate 25 representative designs in a four-dimensional design space. The root and tip installation angles and the root and tip sweep angles are selected as engineering-relevant design variables. Based on the CFD evaluations, quadratic response surface models are constructed to approximate the nonlinear relationships between blade-setting parameters and the key performance indicators, including volumetric flow rate (*Q*), static pressure (*P*), and efficiency (η).^22–24^ Although “RSM+MOGA” has been widely applied, the present work strengthens its engineering use in three aspects. First, RSM is not only used as a surrogate for accelerating optimization but also utilized to quantify sensitivity and coupling effects among variables. This improves the interpretability of how blade root/tip settings affect *Q*, *P*, and η. Second, the multiobjective formulation is constructed to reflect practical cooling fan requirements. It emphasizes a loss-reduction-oriented improvement, i.e., efficiency enhancement under a nearly unchanged flow rate and pressure. The resulting Pareto set is further interpreted to make the compromise mechanism explicit, and a Pareto Front Index (PFI) is adopted for solution ranking in the subsequent analysis. Third, representative Pareto optimal designs are verified and explained through postoptimization diagnostics, including flow-field analysis and a noise-spectrum assessment as an acoustic response check. Overall, this framework provides a transparent route from surrogate modeling to Pareto decision-making and physical interpretation, and it can be extended to the performance-oriented design of rotating machinery in thermal management systems.

## 2 Methodology

### 2.1 Governing Equations and Turbulence Model

The fluid motion obeys the fundamental laws of conservation. In the numerical simulation of the cooling fan flow field, the primary emphasis is placed on aerodynamic performance indicators such as volumetric flow rate, static pressure, and efficiency. Consequently, temperature gradients and large-scale energy conversions can be neglected. Therefore, this investigation employs the Reynolds-averaged Navier–Stokes (RANS) equations, comprising the mass and momentum conservation equations, together with an appropriate turbulence model for flow-field analysis, without the necessity of solving the energy equation.^25^ In computational fluid dynamics, the Finite Volume Method (FVM) is a widely employed discretization technique, offering advantages not only in formulating the governing equations but also in effectively utilizing computational grids.^26^ In essence, the core principle of the FVM is that within a fixed control volume, the rate of mass change equals the net flux of fluid through the three orthogonal directions (*x*, *y*, *z*). Accordingly, the mass conservation equation can be written as

1 $$\frac{\partial \rho }{\partial t}+\nabla \cdot \left(\rho \mathrm{u⃗}\right)=0$$

where ρ represents the fluid density, *u⃗* is the fluid velocity vector, and ∇(*ρu⃗*) denotes the mass flux of the fluid.

Momentum conservation equation, also known as the Navier–Stokes equation, governs the motion of fluid particles under external forces. When external forces are mainly body forces (e.g., gravity) and surface forces (e.g., pressure and viscous stress), the rate of momentum change per unit volume of the fluid is equal to the total external forces acting on that region. This equation has been widely employed in numerical simulations of cooling fans and turbulent flows.^27^ The momentum conservation equation in tensor form can be written as

2 $$\frac{\partial \left(\rho \mathrm{u⃗}\right)}{\partial t}+\nabla \cdot \left(\rho \mathrm{u⃗}⊗\mathrm{u⃗}\right)=-\nabla p+\nabla \cdot \left(\mu \left(\nabla \mathrm{u⃗}+{\left(\nabla \mathrm{u⃗}\right)}^{T}\right)\right)+\rho \mathrm{f⃗}$$

where *u̇⃗* is the velocity vector, *p* is the pressure, μ is the dynamic viscosity, *f⃗* represents body forces (e.g., gravity), ∇(*ρu⃗*⊗*u⃗*) denotes the inertial term, and ∇(μ­(∇*u⃗*+(∇*u⃗*))^*T*^) is the viscous term.

However, in practical engineering applications, such as the complex high-speed flow environment within automotive cooling fans, the flow field generally exhibits strong turbulence. Direct numerical simulation (DNS) of the instantaneous Navier–Stokes equations requires extremely fine grid resolution and small time-steps, leading to prohibitive computational costs. To achieve a balance between computational feasibility and predictive accuracy, this study adopts the Reynolds averaging approach to the momentum conservation equations, thereby formulating the Reynolds-Averaged Navier–Stokes (RANS) equations.^28^ The fundamental concept of Reynolds-averaged momentum conservation equations involves decomposing instantaneous physical quantities into the sum of time-averaged values and fluctuating components

3 $$\phi \left(x,t\right)=\mathrm{\phi ̅}\left(x\right)+\phi^{’}\left(x,t\right)$$

Substituting this decomposition into the original momentum equations and applying time averaging yields the following form of Reynolds-averaged momentum equations

4 $$\frac{\partial \left(\mathrm{p̅}\overset{®}{u_{i}}\right)}{\partial t}+\frac{\partial \left(\mathrm{p̅}\overset{®}{u_{i}}\overset{®}{u_{j}}\right)}{\partial x_{j}}=-\frac{\partial \mathrm{p̅}}{\partial x_{i}}+\frac{\partial }{\partial x_{j}}\left[\mu \left(\frac{\partial \overset{®}{u_{i}}}{\partial x_{j}}+\frac{\partial \overset{®}{u_{j}}}{\partial x_{i}}\right)\right]-\frac{\partial }{\partial x_{j}}\left(\mathrm{p̅}\overset{®}{u_{i}^{’}u_{j}^{’}}\right)$$

where $-\mathrm{p̅}\overset{®}{u_{i}^{’}u_{j}^{’}}$ represents the Reynolds stress term, reflecting the additional influence of turbulent fluctuations on the mean flow. As this term introduces new unknowns, turbulence models (such as *k-ε* and *k-ω*) are required for closure, thereby completing the computable turbulence modeling system. In summary, the RANS equations offer a well-balanced compromise between computational efficiency and accuracy, making them widely used in engineering turbulence simulations and providing the theoretical basis for the numerical analysis of the cooling fan flow characteristics in this study.

This research adopts the Shear Stress Transport (SST) *k-ω* turbulence model, where *k* denotes turbulent kinetic energy and ω represents turbulence frequency. The SST model combines the advantages of both *k-ω* and *k-ε* models; it employs *k-ω* near solid boundaries in the near-wall region, enabling more effective characterization of low dissipation rates and turbulent structures within wall layers; in fully developed free-stream/turbulent regions, its behavior aligns with *k-ε*, offering excellent stability and efficiency while demonstrating excellent performance for separated flows and adverse-pressure gradient conditions.^29^ The transport equations for turbulent kinetic energy *k* and turbulence frequency ω are respectively:

This study adopts the Shear Stress Transport (SST) *k-ω* turbulence model, where *k* denotes the turbulent kinetic energy and ω represents the specific dissipation rate. The selection of SST *k-ω* is motivated by the flow characteristics of automotive axial-flow cooling fans in which near-wall loss mechanisms, adverse-pressure gradients on the blade suction side, tip-leakage-induced vortices, and possible local separation can strongly influence pressure, torque, and efficiency. The SST formulation blends the near-wall advantages of the *k-ω* model with the robustness of *k-ε*-type behavior in the outer flow, thereby improving the reliability for separation/adverse-pressure-gradient conditions and reducing sensitivity to free-stream turbulence specification compared with the standard *k-ω* model. Consequently, SST *k-ω* has become a commonly adopted baseline RANS closure for turbomachinery-related flows and engineering applications similar to the present fan configuration.^30,31^ The transport equations for turbulent kinetic energy *k* and specific dissipation rate ω are, respectively, as follows.

5 $$\frac{\partial \left(\rho k\right)}{\partial t}+\frac{\partial }{\partial \chi_{j}}\left(\rho ku_{j}\right)=P_{k}-D_{k}+\frac{\partial }{\partial \chi_{i}}\left[\left(\mu +\frac{\mu_{t}}{\sigma_{k}}\right)\frac{\partial k}{\partial x_{i}}\right]$$

6 $$\frac{\partial \left(\rho \omega \right)}{\partial t}+\frac{\partial }{\partial x_{i}}\left(\rho \omega u_{i}\right)=C_{\omega }P_{\omega }-\beta_{\omega }\rho \omega^{2}+\frac{\partial }{\partial x_{i}}\left[\left(\mu +\frac{\mu_{t}}{\sigma_{k}}\right)\frac{\partial \omega }{\partial x_{i}}\right]+2\rho \left(1-F_{1}\right)\sigma_{\omega^{2}}\frac{1}{\omega }\cdot \frac{\partial k}{\partial x_{i}}\cdot \frac{\partial \omega }{\partial x_{i}}$$

7 $$\mu_{t}=\frac{\alpha_{1}k}{\max\left(\alpha_{1}\omega ;SF_{2}\right)}$$

where *P_k_*, *P_ω_* are turbulence production terms; *D_k_* is the turbulence dissipation term; σ*_k_* and σ_*ω2*_ are the Prandtl numbers for turbulent kinetic energy *k* and turbulent frequency *ω*, respectively; *F*_1_ and *F*_2_ are blending functions; *S* is the shear strain rate; and *C_ω_*, β*_ω_*, and α_1_ are model constants.^32^

### 2.2 Parametric Fan Modeling

A reasonable aerodynamic model is the foundation for studying the aerodynamic performance and noise characteristics of the cooling fan. This study employs CFturbo software for parametric modeling. During the modeling process, the initial design point (design point) is first established, including parameters such as *Q*, *P*, and rotational speed (*n*), to estimate the main geometric dimensions. Subsequently, the preliminary design is refined and adjusted based on the internal empirical functions/curve library of the CFturbo to balance target performance and manufacturability. The final fan geometry and three-dimensional (3D) model are shown in Figure 1a, and the detailed key parameters are shown in Table 1.

**Figure 1:**
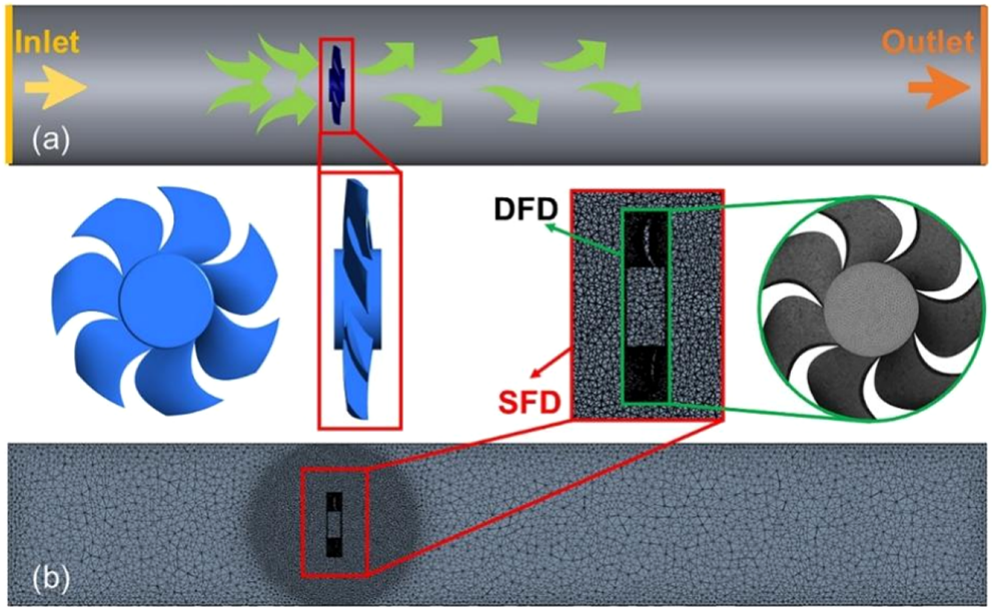
(a) The overall schematic diagram of fan flow-field system, the inset demonstrates the 3D model diagram of the designed fan, and the inlet and outlet are marked by yellow and orange, respectively; (b) mesh configuration in finite element calculation; the inset explains the dynamic fluid domain (DFD) and static fluid domain (SFD), respectively.

**Table 1:** Designed Impeller Diameter (*D*_i_, mm), Impeller Width (*W*_i_, mm), Blade Number (*N*), Root Installation Angle (*γ*_0_), Tip Installation Angle (*γ*_1_), Root Chord Length (*L*_r_, mm), Top Chord Length of Leaves (*L*_l_, mm), Root Sweep angle (*ψ*_0_), and Tip Sweep Angle (*ψ*_1_) of the Investigated Fan

parameters	value	parameters	value
*D*_i_	408	*L*_r_	91
*W*_i_	20	*L*_l_	109
*N*	7	ψ_0_	31.2°
γ_0_	41.6°	ψ_1_	22.0°
γ_1_	10.5°	wing type	NACA65–010

### 2.3 Numerical Simulation Setup

In the Design Modeler module of WorkBench Fluent, the fluid domain was divided into dynamic fluid domain (DFD) and static fluid domain (SFD),^33^ which were meshed separately as shown in Figure 1b. For the dynamic fluid domain, the mesh physics preference was set to CFD with the solver configured as Fluent. The body mesh element size was 20 mm, with refinements applied to the blade surfaces of the impeller, the leading-edge size adjusted to 0.5 mm, other blade surfaces adjusted to 1 mm, and hub/interface regions adjusted to 5 mm. The dynamic fluid domain contained 7.37 million grid cells with a minimum cell quality of 0.21 and maximum skewness of 0.80. The static fluid domain dimensions were configured with a diameter of 2*D*, inlet length of 5*D*, and outlet length of 10*D*, where *D* = 410 mm represents the dynamic fluid domain diameter. The mesh physics preference was also set to CFD (Fluent solver) with a body element size of 30 mm and interface size of 5 mm. A spherical refinement region (radius 500 mm, element size 15 mm) was inserted near the dynamic fluid domain boundary, yielding 3.18 million static fluid domain cells with minimal quality 0.22 and maximal skewness 0.79. Before Fluent parameter setup, negative volume checks were performed and rotational speed units are set as rpm. The Multiple Reference Frame (MRF) method was selected, which simplifies the flow field to an instantaneous snapshot of the impeller position, enabling steady-state solutions for inherently unsteady problems. The fan and surrounding areas were defined in a rotating coordinate system, while other regions used stationary coordinates, making the fan stationary relative to itself. Boundary layer meshing was applied to fan surfaces and stationary walls (Figure 2b). For numerical simulation, pressure-inlet and pressure-outlet boundary conditions were implemented. The inlet and outlet planes were placed sufficiently far from the fan to avoid boundary-induced recirculation and to provide a fully developed inflow–outflow for the investigated operating condition. In the converged solutions, no physically meaningful backflow was observed at the pressure boundaries, indicating that the predicted integral performance metrics are not affected by artificial boundary proximity effects. It is noted that, in practical fan systems, upstream/downstream components may introduce inflow distortion or local recirculation; however, such system-level nonuniformities are beyond the scope of the present isolated-fan study and can be incorporated in future work if needed. The coupled algorithm handled pressure–velocity coupling, while second-order upwind schemes discretized the SST *k-ω* turbulence transport equations (turbulent kinetic energy *k* and specific dissipation rate ω) to ensure stability and accuracy.^22^ Convergence criteria were set as 10^–5^ and 10^–6^ for continuity/momentum equations and turbulence equations, respectively, with air density at 1.225 kg/m^3^ and dynamic viscosity at 1.7894 × 10^–5^ Pa·s.

**Figure 2:**
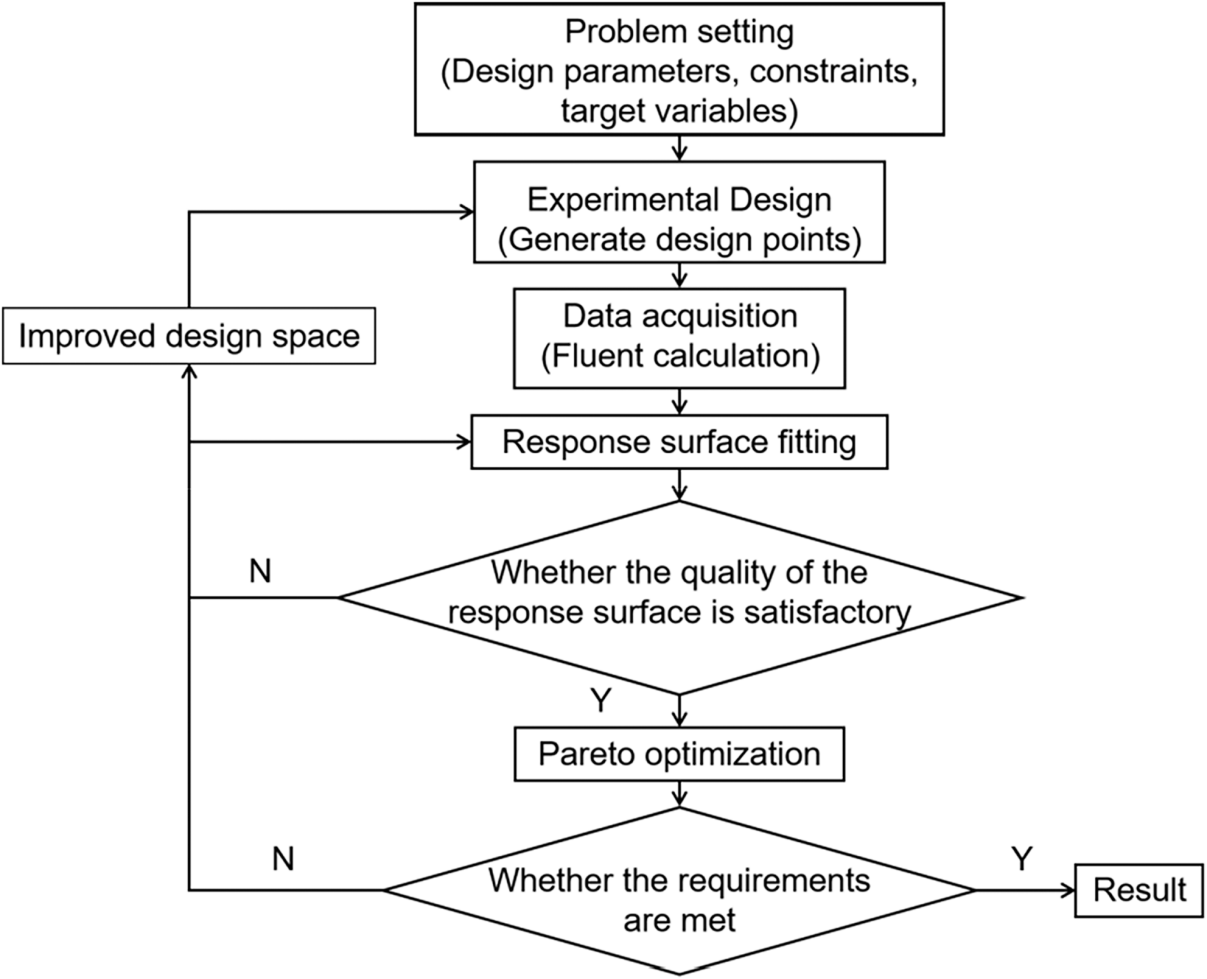
Optimization flowchart of the response surface methodology for this work.

### 2.4 Numerical Verification and Uncertainty Assessment

#### 2.4.1 Convergence Monitoring

All simulations were iterated until the scaled residuals of the governing equations met the prescribed convergence criteria. In addition to residual-based convergence, the integral performance quantities, including volumetric flow rate *Q*, static pressure *P*, and shaft torque *T*, were monitored throughout the iterations to ensure that their values reached a stable plateau. Only the converged solutions with stabilized integral quantities were used for subsequent surface-response construction and optimization analyses.

#### 2.4.2 Grid Independence Verification

A grid-independent study was conducted to assess the numerical consistency of the predicted integral performance quantities. Three systematically refined meshes (coarse/medium/fine) were generated by uniformly adjusting the global and local mesh sizes while keeping the same meshing strategy (i.e., refined blade-surface and leading-edge regions and consistent interface treatment). All three meshes were simulated under the same operating conditions and numerical settings. The resulting volumetric flow rate *Q*, static pressure *P*, shaft torque *T*, and efficiency η are summarized in Table 2.

**Table 2:** Numerical Verification via Grid Independence and Turbulence-Model Sensitivity Check at the Design Operating Condition^a^

case	total cells (×10^6^)	*Q* (m^3^/s)	*P* (Pa)	*T* (N·m)	η (%)
coarse	4.663	2.3029	11.693	0.6887	18.668
medium	10.578	2.2806	11.443	0.6805	18.312
fine	19.177	2.2764	11.396	0.6784	18.258
RNG *k*-*ε* (turbulence model check)	10.578	2.1902	10.557	0.6933	15.925

a The RNG k-ε case is computed on the medium mesh and is included only for turbulence-model sensitivity assessment; it is not part of the mesh refinement sequence

As shown in Table 2, the coarse mesh exhibits noticeable deviations compared with the fine mesh with differences of 1.16% in *Q*, 2.61% in *P*, 1.52% in *T*, and 2.25% in η. In contrast, the medium mesh results are much closer to the fine-mesh predictions, with differences limited to 0.18% *Q*, 0.41% *P*, 0.31% *T*, and 0.30% in η. These results indicate that the medium mesh provides mesh-independent predictions for the integral performance metrics considered in this study while maintaining reasonable computational cost. Therefore, the medium mesh was adopted for all subsequent parametric CFD simulations used to construct the response surface models and to evaluate the Pareto optimal designs.

#### 2.4.3 Turbulence-Model Sensitivity Assessment

To assess the numerical uncertainty associated with turbulence closure, a turbulence-model sensitivity assessment was performed on the medium mesh under the same operating conditions and boundary settings. In addition to the baseline SST *k*-ω model adopted throughout this work, the RNG *k*-*ε* model was also tested. The corresponding results are listed in Table 2 for comparison.

Compared with SST *k*-ω on the medium mesh, RNG *k*-ω predicts lower *Q* and *P* and yields a noticeably lower η, while *T* shows a small difference. Specifically, relative to SST *k*-ω, RNG *k*-*ε* results in decreases of 3.97% in *Q* and 7.74% in *P*, and a decrease of 13.03% in η, whereas *T* increases by 1.88%. This comparison indicates that turbulence-model selection can influence the absolute performance levels for the present fan configuration. To maintain internal consistency in surrogate model construction and multiobjective optimization, all parametric CFD samples and Pareto optimal evaluations were therefore computed using the same baseline turbulence model (SST *k*-ω). The absolute predictions may also depend on boundary-condition specification; this is treated as a limitation of the present steady RANS setup.

## 3 Optimization Framework

### 3.1 Design Variables and Performance Indicators

Our work employs the Response Surface Methodology (RSM) to design and optimize parameters for axial-flow fans under normal operating conditions. CFD-driven performance evaluation and optimization of axial-flow fans has been actively investigated in recent years.^34^ However, for engineering design with limited CFD budgets, surrogate-assisted workflows are beneficial for improving the optimization efficiency and interpretability. RSM is a method based on experimental design theory, which constructs response surface models of objective and constraint functions by conducting tests at specified design point sets, thereby predicting response values at nontested points. The optimization process of RSM used in this study is shown in Figure 2, primarily including problem definition, experimental design, response surface fitting and Pareto optimal solution search.

When design improvements are implemented, the first step is to determine the design parameters for optimization. The critical performance indicators of cooling fans include *Q*, *P*, η, power consumption, and noise level. The fan design requirements involve ensuring sufficient airflow, appropriate static pressure, low power consumption, and high efficiency.^35^ The fundamental design principle is to maximize fan efficiency and minimize noise while meeting engine cooling demands. Considering these factors, this study selects blade root and tip installation angles as well as sweep angles^36,37^ as experimental factors for optimization design. To quantitatively evaluate the flow performance of the investigated axial-flow fan, *Q*, *P*, and η are selected as target variables. The *Q* (m^3^/s) is calculated as

8 $$Q=A\cdot V_{\mathrm{avg}}$$

where *A* is the outlet area of the flow domain (m^2^) and *V*_avg_ is the average velocity at the outlet cross-section (m/s). The *P* is typically defined as the static pressure difference between the outlet and inlet cross-sections

9 $$P=P_{\mathrm{out}}-P_{\mathrm{in}}$$

where *P*_out_ and *P*_in_ show the static pressure at the outlet and inlet cross-sections (Pa), respectively. The η is defined as the ratio of the effective power to the input power

10 $$\eta =\frac{Q\cdot p}{T\cdot \omega }$$

where *T* denotes the fan shaft torque (N·m) and ω is the angular velocity (rad/s). For these target variables, higher *Q* and *P* values with lower *T* indicate better output performance of the investigated fan.

### 3.2 Experimental Design and Surrogate Modeling

We use the Latin Hypercube Sampling (LHS) strategy to generate experimental sample points within the design variable ranges.^38^ As shown in Figure 3a, LHS is a stratified random sampling-based experimental design method that enforces a controlled sample point distribution. This approach prevents localized oversampling, thereby eliminating the risk of missing optimal points while ensuring uniform distribution of samples across the independent variable design space and minimizing intervariable correlations.^39^ Compared with traditional orthogonal arrays or central composite designs, LHS demonstrates superior space-filling capability in high-dimensional spaces, often achieving more representative data distributions with fewer samples. This makes it particularly suitable for modeling nonlinear, multifactorial, and complex systems.^40^ In this study, considering the four-dimensional design space, 25 representative sample points were generated using LHS to construct response surface models for performance metrics, including volumetric flow rate, static pressure, and efficiency, providing data support for subsequent optimization analysis.

**Figure 3:**
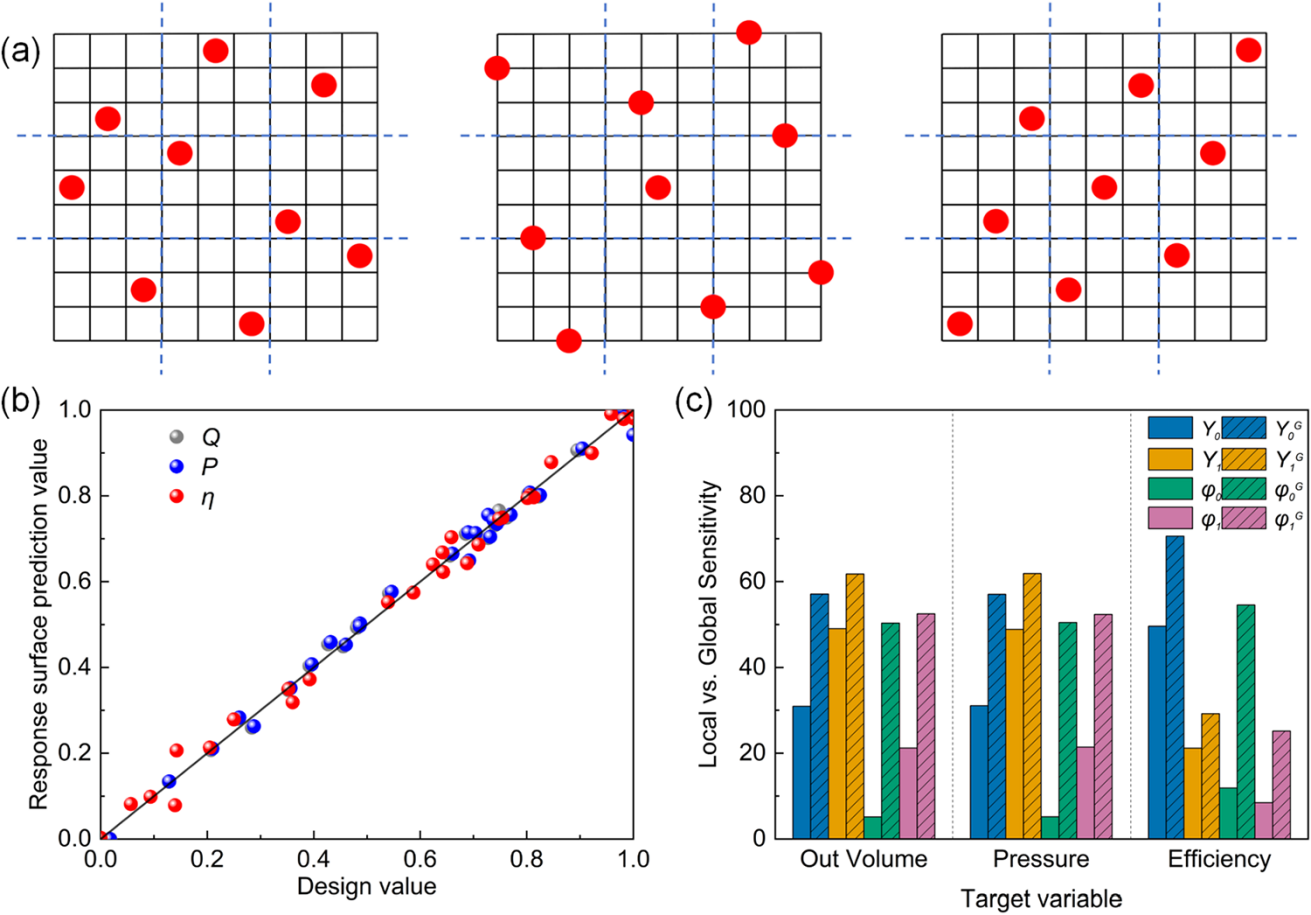
(a) Schematic diagram of sample space for the Latin hypercube sampling design method; (b) goodness-of-fit of the target parameter between the designed and predicted values; and (c) distribution of local and global sensitivities of the target parameter.

### 3.3 Sensitivity Analysis

Response surface fitting establishes the relationship between target variables and design variables through functional approximations based on experimental design points. The quadratic polynomial fitting is used by^41^

11 $$\overset{-}{z}=\beta_{0}+\sum_{i=1}^{n}\beta_{i}y_{i}+\sum_{i=1}^{n}\sum_{j=1}^{n}\beta_{\mathrm{ij}}y_{i}y_{j}$$

where z̅ is the fitted value of the target variable, *n* represents the number of design points, *y*_*i*_ corresponds to the *i*-th design point, and β_0_, β, β_*ij*_ are regression coefficients. To quantitatively evaluate the accuracy of the fitted response surface, the prediction capability of the model was assessed using the coefficient of determination *R* ^2^ and root-mean-square error σ_RMSE_ ^42^

12 $$R^{2}=1-\frac{\sum_{i=1}^{n}{\left(z_{i}-\overset{-}{z_{i}}\right)}^{2}}{\sum_{i=1}^{n}z_{i}^{2}-\sum_{i=1}^{n}\overset{-}{z_{i}^{2}/n}}$$

13 $$\sigma_{\mathrm{RMSE}}=\sqrt{\frac{{\sum_{i=1}^{n}\left(z_{i}-\overset{-}{z_{i}}\right)}^{2}}{n}}/\frac{\sum_{i=1}^{n}z_{i}}{n}$$

where *z*_*i*_ is the observed value at the design point, and *z̅*_*i*_ is the fitted value from the response surface function. Based on the definitions of *R* ^2^ and σ_RMSE_, the closer *R* ^2^ is to 1 and the closer σ_RMSE_ is to 0, the higher the accuracy of the response surface fit. Figure 3b presents the goodness-of-fit plot of the quadratic polynomial response surface, constructed from the design points in the sample space, to assess the consistency between the model’s predicted values and the actual sample data.^43^ In Figure 3b, the horizontal axis represents the numerically calculated values of the target variables at the design points, while the vertical axis represents the predicted values of the target variables from the response surface. It is evident that the predicted values of the response surface for different target variables vary with the observed values at the design points, generally exhibiting a linear relationship with a slope of 1, indicating the high accuracy of the response surface fit. As shown in Table 3, the quadratic RSMs show high predictive accuracy for *Q*, *P*, and η, indicating small fitting errors within the sampled design domain. Since all candidate designs considered in the subsequent optimization are restricted to the same bounded variable ranges used for surrogate construction, the surrogate is applied in an interpolative manner rather than extrapolation. Therefore, the influence of surrogate fitting errors on subsequent optimization-based comparisons is expected to be limited at the current accuracy level.

**Table 3:** Fitted Evaluation Results

target variable	*R* ^2^	σ_RMSE_
*Q*	0.995	0.019
*P*	0.995	0.002
η	0.991	0.069

Figure 3c shows the partial variance comparison of the independent variables, illustrating their local sensitivity to the target variables and highlighting the relative importance of each variable to *Q*, *P*, and η. The sensitivity index is defined as

14 $$S_{i}=\frac{V_{i}}{V}$$

where *V*_*i*_ represents the influence of an independent variable on the target variable, *V* denotes the impact of all independent variables on the target variable, and *S*_*i*_ indicates the local sensitivity.

Figure 3c compares the local (solid bars) and global (hatched bars) sensitivity indicators of the four design variables for the three target responses, *Q*, *P*, and η. For *Q* and *P*, the local sensitivities are dominated by the installation angles: the tip installation angle contributes approximately 49.0% and 48.9%, respectively, whereas the root installation angle shows a smaller local contribution. In terms of η, the local sensitivity is primarily governed by the root installation angle (about 49.60%), indicating that the efficiency is most responsive to the root setting near the reference design.

Across all three responses, the dominant-variable ordering inferred from the global indicator is broadly consistent with the local analysis, while the sensitivity magnitudes differ noticeably between the two metrics. This gap implies that, within the investigated ranges, a local measure alone may not fully represent full-space response variability and that coupling/nonlinear effects can contribute to the observed differences. Consequently, installation angles remain the primary levers for performance tuning, whereas sweep angles generally play a secondary fine-adjustment role.

## 4 Results and Discussion

### 4.1 Response Surface Methodology

To further investigate the influence patterns of design variables on fan performance, we plot response surfaces of *Q*, *P*, and η as functions of the design variables based on the response surface model. Three representative figures are selected for explanation: First, Figure 4a–r illustrates the trends of the target variables with changes in design parameters. It is evident that γ_1_ has the most significant effect on enhancing *P*; as it increases, static pressure steadily rises, reflecting that flow control in the blade tip region is crucial for the compression capability of the fan. By comparison, γ_0_ has a weaker impact on *P*, playing a role only within a small range of angles. Thus, the primary optimization strategy for *P* remains to improve the rationality of the tip installation angle. Additionally, Figure 4n shows the variation of *Q* with γ_1_ and ψ_1_, which can be seen that *Q* is highly sensitive to the tip installation angle. As γ_1_ increases, *Q* significantly improves, indicating that the design of the tip attack angle directly determines the airflow acceleration effect. By comparison, the variation along the ψ_1_ direction is relatively small, with the surface being overall smooth, suggesting that ψ_1_ optimizes the airflow path and enhances *Q*. Thus, within the designed space, γ_1_ is the dominant parameter determining the volumetric flow rate level, while the influence of ψ_1_ is relatively secondary. Finally, Figure 4l demonstrates the response relationship of η with γ_1_ and ψ_0_. The results indicate that the efficiency is also sensitive to changes in γ_1_. Within the current design range, an increase in γ_1_ generally leads to improved efficiency, but the surface tends to flatten at larger values, indicating a diminishing effect. The influence of ψ_0_ manifests as a region of higher efficiency within a moderate range, while values that are too large or too small cause efficiency to drop to lower levels, suggesting that ψ_0_ plays a regulatory role in energy utilization. In summary, γ_1_ exhibits significant influence across all three performance metrics, making it the core parameter for optimization design. The ψ_1_ and ψ_0_ play auxiliary roles in regulating flow losses and efficiency. Reasonable configuration of these parameters helps improve efficiency while enhancing volumetric *Q* and *P*, achieving coordinated optimization across multiple objectives.

**Figure 4:**
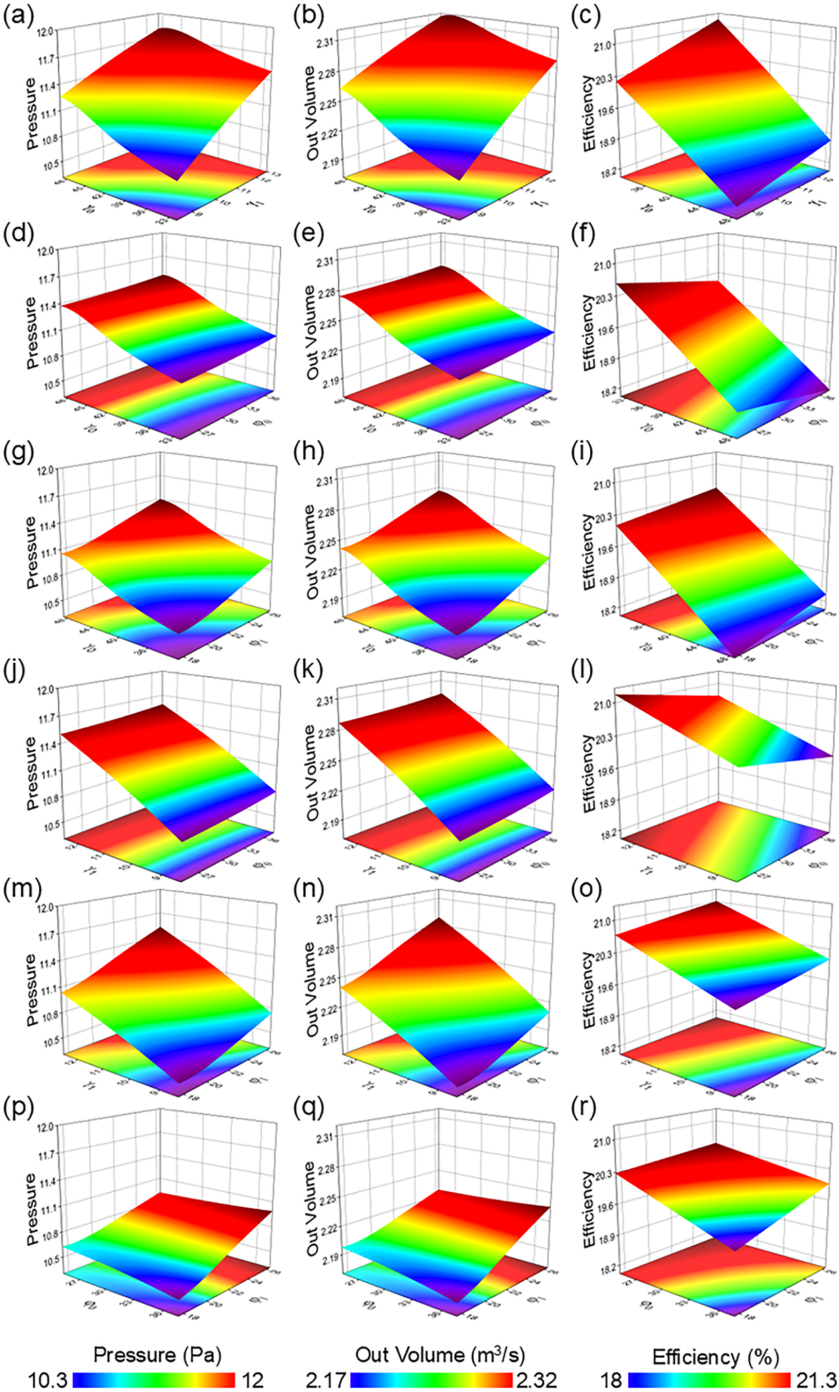
Calculated (a, d, g, j, m, p) static pressure, (b, e, h, k, n, q) volumetric flow rate, and (c, f, i, l, o, r) efficiency of the proposed mode under different critical parameters by (a, b, c) γ_*0*_ and γ_*1*_, (d, e, f) γ_*0*_ and ψ_*0*_, (g, h, i) γ_*0*_ and ψ_1_, (j, k, l) γ_1_ and ψ_*0*_, (m, n, o) γ_1_ and ψ_1_, and (d, e, f) ψ_*0*_ and ψ_1_.

### 4.2 Multiobjective Genetic Algorithm (MOGA)

To simultaneously improve *Q*, *P*, and η, the following multiobjective optimization mathematical model is constructed under design variable constraints, with the Pareto optimal set serving as the criterion for evaluating the solution set^44^

15 $$\{\begin{array}{l} \max\left(Q\right)\leq 1\\ \max\left(P\right)\leq 1\\ \max\left(\eta \right)\leq 1\\ X_{\min}\leq X_{i}\leq X_{\max} \end{array}$$

where *X*_*i*_ is the design variable, *X*_min_ is the lower limit of the design variable, and *X*_max_ is the upper limit of the design variable. In multiobjective optimization, it is impossible to achieve the optimal values of *Q*, *P*, and η simultaneously. The set of all possible solutions constitutes the Pareto optimal solution set, which consists of solutions where the improvement of any one objective variable must come at the expense of other objective variables. It should be noted that the present Pareto search is conducted on the RSM-based surrogate models. Given the high predictive accuracy of the surrogate within the bound design space, the surrogate fitting errors are small relative to the objective variations among Pareto candidates. Therefore, the overall shape and dominant trade-off trends of the Pareto front are unlikely to be altered by the surrogate uncertainty at the current accuracy level. For solutions with extremely close objective values, minor ranking changes within the surrogate error level may occur; such solutions can be regarded as near-equivalent from an engineering perspective. To search for compromise solutions between objectives and maintain the diversity and uniform distribution of the solution set, a mature multiobjective genetic algorithm (MOGA) framework for solution is explored.^45^ Figure 5 shows the basic optimization process of MOGA. To generate the initial population and ensure low-discrepancy coverage of the design space, initial samples are generated using Shifted Hammersley Sampling (SHS). SHS belongs to the category of low-discrepancy sequence methods, which generally outperform purely random sampling or simple Latin Hypercube Sampling (LHS) when surrogate models are constructed and initial samples. This study employs SHS to generate initial samples (with the sample size set to 1000 *N* = 4000),^46^ where *N* is the number of independent variables, and then calculate the volumetric flow rate, static pressure, and efficiency. If the termination criterion is met, then the termination population is generated; if not, processes such as fitness assignment, genetic operations, and parent insertion are performed to obtain the next generation population until the termination criterion is satisfied.

**Figure 5:**
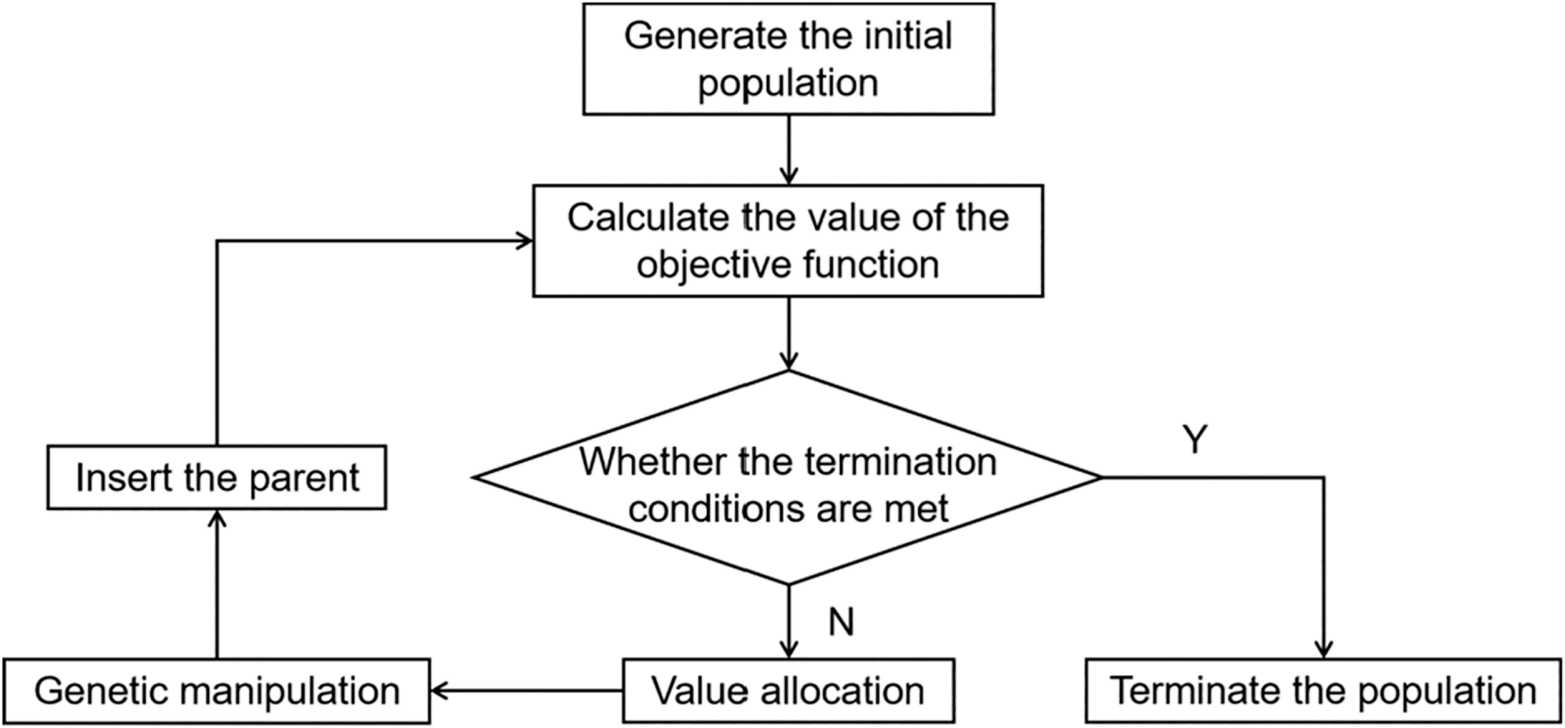
Process diagram of MOGA in our work.

### 4.3 Optimization Outcomes

To reduce unnecessary iterations and objectively determine whether the algorithm has converged, this study introduces two convergence criteria: Maximum Allowable Pareto Percentage (MAPP) and Convergence Stability Percentage (CSP). When the proportion of Pareto points reaches the preset MAPP or when the changes in the population mean and variance meet the CSP setting, the algorithm is considered to have converged and stops iterating.^47^ The stability percentage is calculated based on the mean and variance of the objective function values of the current and previous generations to measure the overall stability of the population. When this stability percentage falls below the preset threshold of CSP (set to CSP = 0.01% in this study), the algorithm is judged to have converged. Its mathematical expression is

16 $$\frac{\left|Y_{i}-Y_{i-1}\right|}{Y_{\max}-Y_{\min}}<\frac{S}{100}$$

17 $$\frac{\left|\sigma_{i}-\sigma_{i-1}\right|}{Y_{\max}-Y_{\min}}<\frac{S}{100}$$

where *S* represents the percentage of convergence stability. *Y*_*i*_ and *Y*_*i*–1_ are the average values of the *i*-th and (*i*–1)-th generation populations, respectively; σ_*i*_ and σ_*i*–1_ are the variances of the *i*-th and (*i*–1)-th generation populations, respectively. *Y*_max_ and *Y*_min_ are the maximal and minimal values of the initial population, respectively. Figure 6a shows the trend of the Pareto percentage and stability percentage with the number of iterations. It is evident that after 28 iterations, the stability percentage reaches 0.0084%, which is less than 0.01%, meeting the convergence criterion. Figure 6b presents the distribution of the Pareto solution set where different colors and sizes represent different Pareto Front Indices. A smaller PFI indicates that the Pareto solution is more desirable. The distribution of solutions in the design space is mainly concentrated near the Pareto front, with relatively sparse distribution in other regions, indicating that during the optimization process, the design variables (installation angle and bending sweep angle) gradually approach the optimal target variables *Q*, *P*, and η. The selected Pareto optimal solutions are given in Table 4.

**Figure 6:**
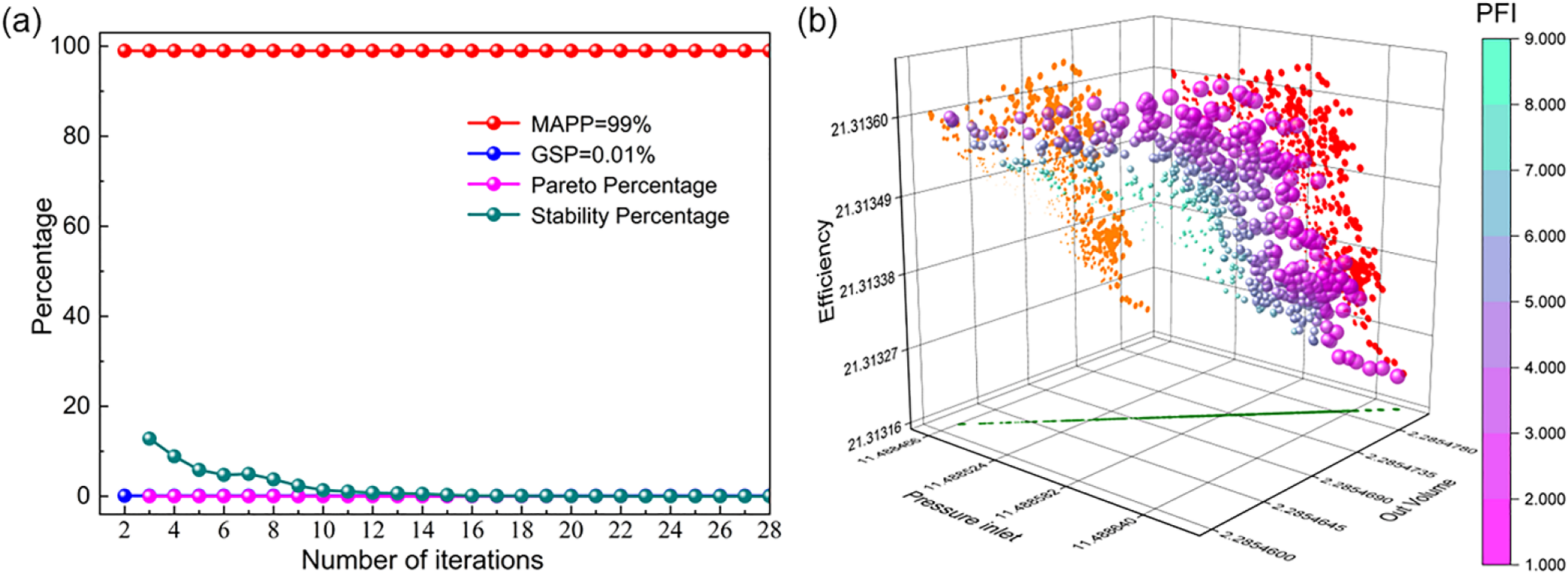
(a) The trend of convergence criterion with the number of iterations; (b) pareto solution set distribution.

**Table 4:** Pareto Optimal Parameters of Root Installation Angle (*γ*_0_, °), Tip Installation Angle (*γ*_1_, °), Root Sweep Angle (*ψ*_0_, °), Tip Sweep Angle (*ψ*_1_, °) of the Fan for Optimal Solution of Target Variable of Flow (*Q*, m^3^/s), Static Pressure (*P*, Pa), and Efficiency (*η*, %)

scheme	γ_0_	γ_1_	φ_0_	φ_1_	*Q*	*P*	_η_
1	32.081	12.576	24.966	26.351	2.2855	11.489	21.313
2	32.08	12.575	24.964	26.352	2.2855	11.488	21.313
3	32.081	12.575	24.961	26.351	2.2855	11.488	21.313

To improve the methodological coherence between the CFD simulations and the subsequent optimization, the final optimal candidates obtained from the RSM-assisted MOGA search were subjected to a posteriori CFD verification. Specifically, the design variable combination corresponding to optimization scheme 1 in Table 4 was reconstructed (while keeping all other geometric features unchanged) and resimulated using the same CFD settings and meshing strategy as those employed for the design of experiments database. Table 5 compares the surrogate-predicted target responses and the re-evaluated CFD results before and after optimization. The optimized design exhibits little change in *Q* and *P* relative to the baseline, while the static efficiency increases by 2.882%. Moreover, the differences between the surrogate predictions and the verified CFD values at the selected optimal point remain small, indicating that the surrogate-based optimization provides reliable guidance and that the obtained optimal solution is numerically consistent with the underlying CFD model.

**Table 5:** Comparison of Target *Q* (m^3^/s), *P* (Pa), and *η* (%) before and after Optimization

	*Q*	*P*	η
before optimization	2.2806	11.443	18.312
after optimization	2.2855	11.394	21.194

### 4.4 Flow-Field Analysis

Figure 7a,b illustrates the velocity vector distribution of the fan under identical operating conditions before and after optimization in this study. The preoptimization image reveals relatively complex internal flow within the air duct, with noticeable low-velocity zones at the blade root and near the hub region, where partial airflow exhibits backflow phenomena. Additionally, irregular vortex structures with chaotic vector directions form near the blade trailing-edge and wall-adjacent regions. These phenomena indicate energy loss as airflow passes through the blades, reducing the overall flow-field stability and transport efficiency. In contrast, the optimized velocity vector distribution demonstrated markedly different characteristics. The overall flow field becomes smoother, with airflow closely adhering to the blade profile along the surface, low-velocity backflow zones significantly reduced, and the large-scale vortex structures effectively suppressed. In particular, near the blade trailing-edge and outlet regions, vector directions become uniform and streamline distribution more continuously, demonstrating more efficient utilization of airflow kinetic energy. These improvements are closely related to optimization of the tip installation angle. After increasing the tip installation angle, the effective angle of attack in the working section of the blade is improved, enhancing the pressure gradient on the suction side of the blade tip region. This promotes better airflow attachment and transport along the blade surface, thereby suppressing the initial flow separation tendency near the blade tip. Simultaneously, the larger installation angle improves the pressure matching between the blade tip and the end wall, weakening the end-wall effect and reducing the strong radial migration of flow near the end wall. More importantly, the optimized load distribution at the blade tip becomes more reasonable, significantly diminishing the pressure difference-driven three-dimensional secondary flows (including end-wall vortices, horseshoe vortices) near the end wall. The reduction in secondary flow intensity directly leads to a decrease in vortex structures in the near-wall region and a reduction in the scale of trailing-edge shedding vortices, resulting in a more orderly flow throughout the passage. Comparative analysis clearly shows that flow separation phenomena at the blade root and trailing-edge regions are substantially mitigated after optimization, with backflow and vortex ranges significantly reduced, resulting in a more stable and organized overall flow field. These improvements not only reduce energy loss but also enhance aerodynamic efficiency, consistently aligning with the performance enhancement trend observed in numerical calculations, thereby validating the effectiveness of structural parameter optimization.

**Figure 7:**
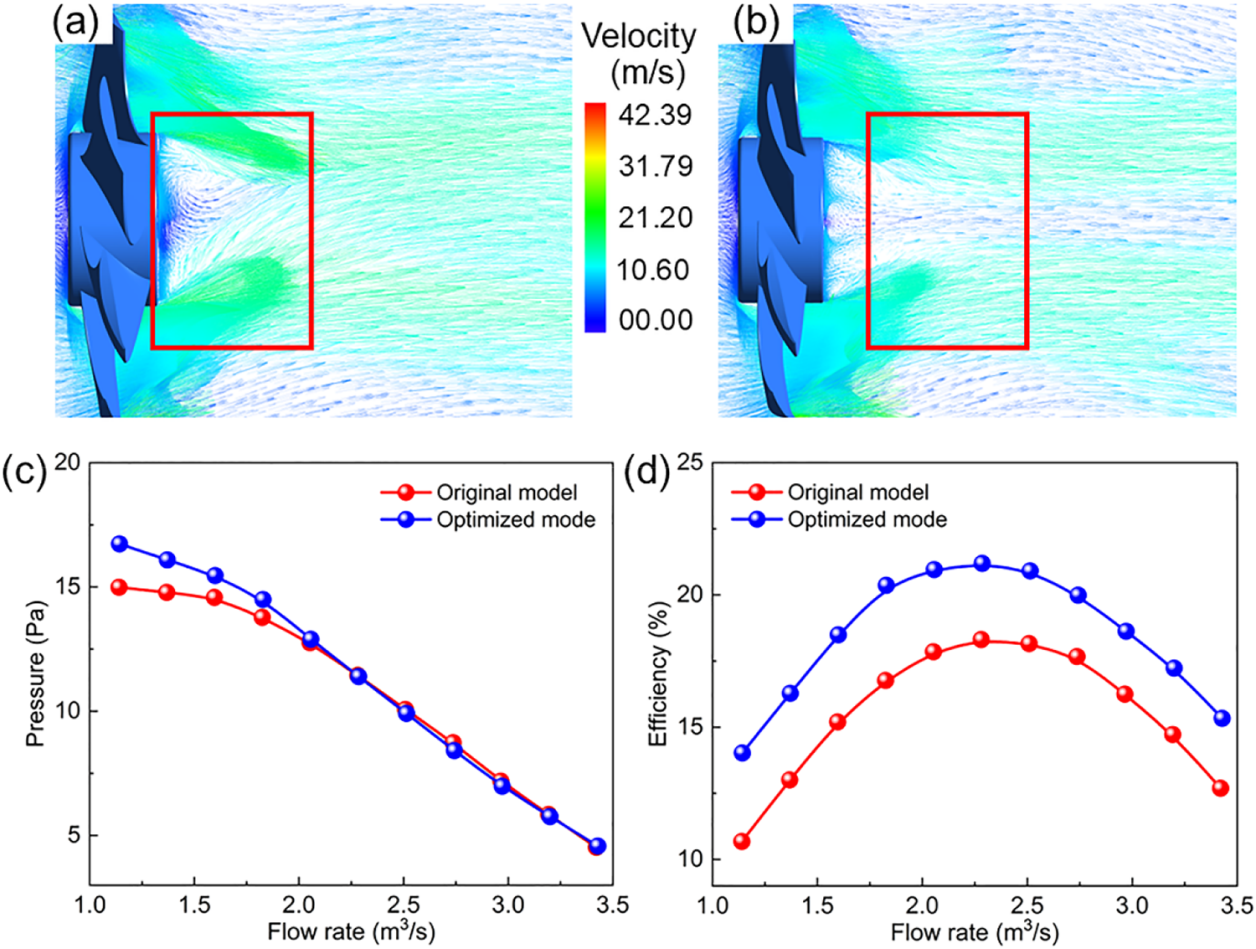
Calculated velocity vector distribution of (a) original and (b) optimized models; the output (c) pressure and (d) efficiency of the original and optimized model under different volumetric flow rate.

Figure 7c,d shows the optimized *Q*–*P* curve and *Q*–η curve of the axial-flow compared with that of the initial one, respectively. The *Q*–*P* indicate that the overall trends before and after optimization are nearly overlapping, with minimal differences in the medium-to-high volumetric flow rate regions, suggesting that the optimization did not significantly alter the compression capability and static pressure characteristics of the fan. These results highlight that the primary effect of the optimization was focused on efficiency improvement, while maintaining the original static pressure performance. The *Q*–η curves exhibit notable differences, with the postoptimization curve shifting upward overall. Efficiency is higher across the entire volumetric flow rate range compared to the original design, and the peak efficiency is significantly improved, while the peak position remains largely unchanged. This efficiency improvement can be attributed to the flow mechanisms induced by the increased tip installation angle. After optimization, the effective angle of attack near the blade tip is moderately increased, leading to a more reasonable load distribution around the design point. This reduces the local energy dissipation caused by trailing-edge separation and uneven pressure differences between the suction and pressure surfaces. Simultaneously, the improved pressure gradient near the end wall weakens the end-wall secondary flow structures, resulting in a significant reduction in the three-dimensional losses. The attenuation of secondary flows and end-wall vortices yields a more uniform velocity distribution at the outlet section and lowers the passage energy loss, which is directly reflected in the overall elevation of the fan efficiency curve. Overall, the optimized fan achieves an improvement the in overall efficiency without compromising static pressure performance, validating the effectiveness and engineering application value of the optimization method for energy efficiency enhancement.

### 4.5 Aerodynamic Noise-Spectrum Characteristics before and after Optimization

The sound pressure level (SPL) spectra of the original and optimized models are compared in Figure 8 to examine the acoustic response associated with the flow-field modification induced by geometric optimization. It should be noted that the present optimization primarily targets aerodynamic objectives, and the noise metric is not included as an explicit objective. Therefore, the spectral comparison is intended to characterize frequency-dependent changes in SPL rather than to imply a targeted broad-band noise reduction.

**Figure 8:**
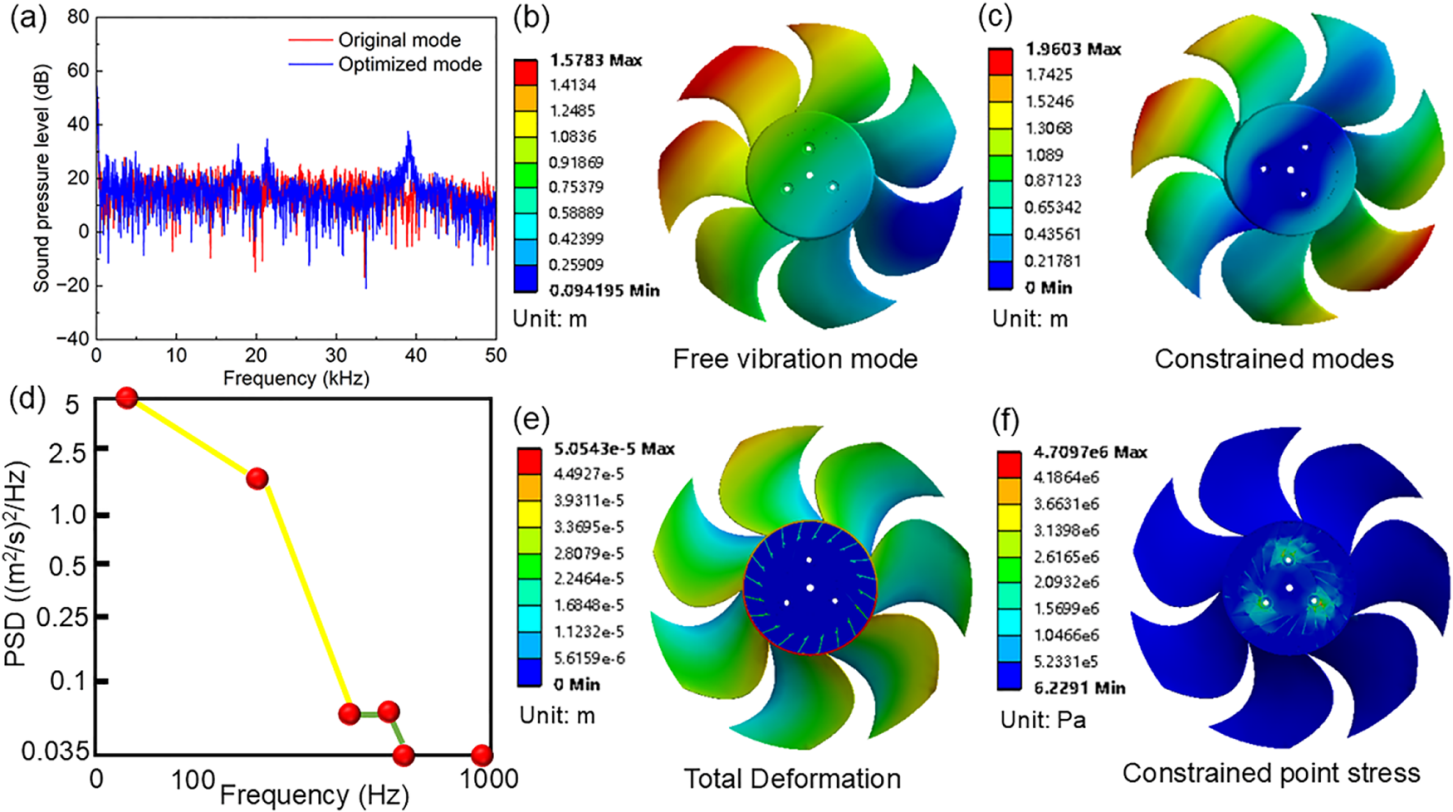
(a) The calculated sound pressure level spectra of the original and optimized model; (b) and (c) represent the deformation of optimized model under the vibration modes of the seventh order free mode and the first-order constrained mode; (d) the acceleration power spectral density (PSD) characterizes from QCT773 standard; (e) and (f) represent the total deformation and stress of the optimized models under PSD.

As shown in Figure 8a, the two spectra exhibit band-dependent differences, with the most apparent variations occurring in the low to mid frequency range. In the low-frequency region (below approximately 125 Hz), the optimized model shows an SPL slightly higher than that of the original model. In contrast, a distinct attenuation is observed in the mid to low-frequency band around 160–220 Hz, where the optimized spectrum remains consistently lower. At higher frequencies, the differences become less monotonic: an overall reduction tendency can be identified over an intermediate band on the order of 400–700 Hz, whereas a localized enhancement is observed in a higher-frequency band (approximately 0.8–3.2 kHz). Beyond several kilohertz and up to the upper frequency limit shown (50 kHz), the two spectra are generally closer, although band-dependent deviations still exist. Overall, Figure 8 indicates that the aerodynamic improvement does not directly correspond to a uniform decrease in SPL across the entire frequency range; instead, it is accompanied by a redistribution of acoustic energy among different frequency bands. This observation highlights the nontrivial coupling between the aerodynamic performance and acoustic behavior.

Then, to verify the practicality of the designed leaf shape, we further studied the vibrational characteristics and strength. We combined the designed blade shape with the current mainstream installation method and used an Ansys instrument to perform grid division on it. Figure 8b shows the deformation pattern of the seventh-order free vibration mode in which the blade deformation is dominated by global bending without mechanical constraints, exhibiting relatively large modal displacement amplitudes. Figure 8c presents the deformation distribution of the first-order constrained mode, where the imposed boundary constraints effectively suppress rigid-body motion and significantly alter the modal shape, resulting in reduced displacement localization and enhanced structural stiffness. The acceleration power spectral density (PSD) specified according to the QCT773 standard, shown in Figure 8d, is applied as the excitation input for the vibration response analysis, which characterizes the frequency-dependent distribution of vibrational energy under random excitation. Figure 8e demonstrates the total deformation of the optimized fan subjected to the prescribed acceleration PSD, which shows the maximal deformation remains limited and spatially smooth, indicating favorable vibration resistance. Considering that the fan is fabricated from PA66 reinforced with 30% glass fiber (PA66+GF30) with a tensile strength of 175 MPa, the deformation levels fall well within the allowable elastic range, confirming adequate structural rigidity under stochastic vibration loading, as shown in Figure 8f with maximal stress of 4.71 MPa.

## 5 Conclusion

This study integrates response surface methodology with a multiobjective genetic algorithm to optimize the structural parameters of an automotive axial-flow cooling fan, achieving a significant efficiency improvement from 18.31% to 21.19% while maintaining stable flow rate and static pressure. Sensitivity analysis identifies the installation angle as the dominant factor affecting performance, and flow-field results confirm the reduced separation and vortex intensity, leading to enhanced aerodynamic stability. Acoustic analysis shows that efficiency gains mainly redistribute noise energy across frequency bands rather than uniformly reducing noise levels, underscoring the complex aero-acoustic coupling. Overall, the proposed framework proves effective for aerodynamic performance enhancement with acceptable acoustic behavior and shows promise for broader application in rotating thermal management machinery, although future work should incorporate modal analysis under rotational prestress to ensure structural safety.
